# 511 Characteristics of Inhalation Injury Management at a Referral Burn Center: A Ten-year Experience

**DOI:** 10.1093/jbcr/irac012.142

**Published:** 2022-03-23

**Authors:** Mack Drake, Stefan Leichtle, C Todd Borchers, Naushin S Ali, Nayla Labban, Michael J Feldman

**Affiliations:** VCU Health, Richmond, Virginia; VCU Health, Richmond, Virginia; VCU Health, Richmond, Virginia; VCU School of Medicine, Richmond, Virginia; VCU School of Medicine, Richmond, Virginia; VCU Health, Richmond, Virginia

## Abstract

**Introduction:**

Variability exists in the diagnosis and treatment of upper airway and inhalation injury (UAII). Institutional algorithm at our ABA-verified burn center in part necessitates fiberoptic diagnostic laryngoscopy (DL) in patients at risk for UAII. We sought to determine patterns and characteristics of UAII management and complications over a ten-year period.

**Methods:**

A retrospective review of burn admissions between January, 2011 and December, 2020 was performed. 253 patients with concern for UAII underwent DL upon presentation. Patients under age 18 were excluded. 66 patients were excluded for inability to tolerate/refusal/unreported results. Univariate and multivariate analyses were used to determine independent predictors of a positive DL.

**Results:**

169 patients were analyzed. The population was frequently middle-aged, male, and overweight. 81 patients used tobacco and 34 patients had history of COPD. Examination of injury characteristics yielded median ISS of 6. 116 patients (69%) had no or < 1% cutaneous thermal burns. 75 patients had facial soot or singed nasal hairs concerning for UAII. Of 169 patients who underwent DL, DL was positive in 106 (63%). Tracked complications in patients with positive DL yielded few unplanned intubations (n=2, 1%), unplanned ICU admission (n=2, 1%), unplanned extubation (n=4, 2%), VAP (n=1, 1%). Median ICU LOS was 2 days and average hospital LOS was 3 days. Mortality overall was 5% (n=8). Subgroup analysis was used to compare patients with positive versus negative DL. Patients with positive DL were older (54 vs 49 years, p=0.09), primarily male (63% vs 60%, p=0.7), had lower BMI (29 vs 30, p=0.94), were tobacco users (52% vs 41%, p=0.18), and often carried diagnosis of COPD (24% vs 14%, p=0.15). Median ISS was higher in the positive DL group (10 vs 4, p< 0.0001). Cutaneous burns were absent or < 1% (85 vs 58, p=0.05). Presence of facial burns and soot was significantly associated with positive DL (56 vs 19, p< 0.0001, OR 15.9, 95% CI). With positive DL, median ICU LOS, hospital LOS, and mortality were significantly higher (p< 0.0001, p< 0.0001, and p=0.03, respectively).

**Conclusions:**

These findings lend credit to an approach of aggressive diagnosis and subsequent management. Multivariate analysis of factors associated with positive DL highlighted obvious facial burns or soot and ISS (OR 1.1, 95% CI, p=0.01) as most predictive. Though rates of tracked complications were too low for subgroup analysis, no patient with negative DL on admission required unplanned intubation. Patients with positive DL at our institution are placed on an algorithm of ICU admission, 24-hour period of NPO, mucolytic therapy, enhanced pulmonary hygiene, and lung protective ventilatory strategies. Patients with positive DL exhibited longer ICU and hospital LOS and increased mortality.

